# Genetic diversity of *Taenia saginata* (Cestoda: Cyclophyllidea) from Lao People’s Democratic Republic and northeastern Thailand based on mitochondrial DNA

**DOI:** 10.1186/s13071-017-2079-7

**Published:** 2017-03-11

**Authors:** Oranuch Sanpool, Rutchanee Rodpai, Pewpan M. Intapan, Lakkhana Sadaow, Tongjit Thanchomnang, Sakhone Laymanivong, Wanchai Maleewong, Hiroshi Yamasaki

**Affiliations:** 1 Department of Parasitology and Research and Diagnostic Center for Emerging Infectious Diseases, Faculty of Medicine, Khon Kaen University, Khon Kaen, 40002 Thailand; 2 Faculty of Medicine, Mahasarakham University, Maha Sarakham, 44000 Thailand; 3 Centre of Malariology, Parasitology and Entomology, Ministry of Health, Vientiane, Lao PDR; 4 Department of Parasitology, National Institute of Infectious Diseases, Ministry of Health, Labour and Welfare, Tokyo, 162-8640 Japan

**Keywords:** *Taenia saginata*, Beef tapeworm, Genetic diversity, Haplotypes, Distribution, Lao PDR, Thailand

## Abstract

**Background:**

*Taenia saginata* is a tapeworm found in cattle worldwide. Analysis of genetic diversity in different geographical populations of *T. saginata* not only helps to understand the origin, transmission and spread of this organism, but also to evaluate the selection pressures acting on *T. saginata* and how it is responding to them. However, there are few reports of the genetic variability of *T. saginata* populations in different regions of the world, including Lao PDR and Thailand. We report the genetic diversity of *T. saginata* populations in Lao PDR and northeastern Thailand together with sequences of *T. saginata* from other countries deposited in GenBank.

**Results:**

Mitochondrial *cox*1 sequence analysis revealed that 15 and 8 haplotypes were identified in 30 and 21 *T. saginata* isolates from Lao PDR and northeastern Thailand, respectively. Fifty-three haplotypes were identified from 98 sequences. Phylogenetic tree and haplotype network analyses revealed that global isolates of *T. saginata* were genetically divided into five groups (A, B, C1, C2 and D). *Taenia saginata* isolates from Lao PDR and northeastern Thailand belonged to either Group A or B. *Taenia saginata* from western Thailand clustered in groups C1, C2 and D, and populations from the northeast and western Thailand were found to be genetically distinct. *Taenia saginata* isolates in Lao PDR and Thailand were also found to be genetically diverse but the degree of genetic differentiation was low.

**Conclusions:**

*Taenia saginata* populations from Lao PDR and northeastern Thailand are genetically distinct from the population in western Thailand and it is proposed that *T. saginata* has been dispersed by different transmission routes in Southeast Asia.

**Electronic supplementary material:**

The online version of this article (doi:10.1186/s13071-017-2079-7) contains supplementary material, which is available to authorized users.

## Background

Taeniosis is a parasitic zoonosis caused by infection with *Taenia saginata*, *Taenia solium* or *Taenia asiatica* [1, 2]. *Taenia saginata*, known as the beef tapeworm*,* has a global distribution which includes Thailand [3, 4] and Lao PDR [5, 6]. The intermediate host of the parasite is cattle and larval cysticerci are found in the muscle tissue of the host. Humans can become infected by ingestion of a cysticercus in raw or undercooked beef which then develops into an adult tapeworm in the small intestine. The adult tapeworm sheds gravid proglottids filled with eggs, which pass into the environment in feces and are degraded to release the eggs. Once eggs are ingested by the intermediate cattle host, the larval oncospheres in the eggs hatch in the small intestine and migrate to skeletal muscle where they develop into cysticerci which are infectious to humans. Most patients infected with *T. saginata* have an active discharge of gravid proglottids and occasionally present with epigastric pain, nausea, weight loss and poor appetite [2]. Cattle infected with *T. saginata* can cause economic losses in terms of food production [7–10] and trade restriction [11].

Information on the global genetic diversity of *T. saginata* is very limited. Mitochondrial DNA has been used as a genetic marker for detection of intraspecific variation or cryptic species [12], and cytochrome *c* oxidase subunit 1 gene (*cox*1) is a commonly used marker for studying geographical populations of *T. saginata* [4, 13, 14]. Anantaphruti et al. [4] reported that 14 haplotypes were identified from 73 *T. saginata* isolates from northern and northeastern regions of Thailand based on the partial *cox*1 sequences (924 bp). Although the distribution range of *T. saginata* is known for Lao PDR, which borders Thailand [5, 6], no evidence of genetic variation of *T. saginata* has been reported to date.

In this study, we performed molecular-phylogenetic tree and haplotype network analyses based on the complete *cox*1 sequences (1,620 bp) of 51 *T. saginata* isolates from Lao PDR and northeastern Thailand. We also included 47 sequences already deposited in GenBank, to better understand the genetic diversity and character of *T. saginata* populations in these countries.

## Methods

### Parasite samples

The 51 taeniid samples examined in this study were collected in Lao PDR (*n* = 30) and northeastern Thailand (*n* = 21). In Lao PDR, specimens were obtained from patients with taeniosis in Xebungfai District, Khammouane Province, central Lao PDR (17°04′287″N, 104°54′544″E) during a parasitological survey conducted by the Lao Ministry of Health in June 2015 [15]. Patients with taeniid eggs detected in the feces by the Kato-Katz technique [16] were treated with a single dose of praziquantel (40 mg/kg) and saturated magnesium sulfate solution. The dose of praziquantel was increased because taeniosis patients are commonly co-infected with liver fluke (*Opisthorchis viverrini*) in Lao PDR [17]. The proglottids obtained by deworming were washed with normal saline solution, fixed in 70% ethanol, and stored at -20 °C until use. Taeniid proglottids from Thailand were obtained from patients who lived in northeastern regions of Thailand and had been admitted to Srinagarind Hospital, Faculty of Medicine, Khon Kaen University, Khon Kaen, Thailand (2010–2015) and had been stored in 70% ethanol at -20 °C.

### Molecular identification of taeniid parasites

Genomic DNA samples were extracted from individual taeniid proglottids using a DNeasy Blood & Tissue kits (Qiagen, Hilden, Germany) according to the manufacturer’s instructions. The complete *cox*1 gene (1,620 bp) was amplified by PCR using the primers Taenia trnW/F (5′-GTT ATG TTA GAC TAG ATG TTT TCA-3′ for the *Taenia* transfer RNA-Try gene) and Taenia rrnl/R (5′-TCC ACT AAG CAT AAT GCA AAA GGC-3′ for *Taenia* ribosomal RNA large subunit gene) [18]. TaKaRa Ex *Taq* (Hot Start version, Takara Bio, Shiga, Japan) was used as a DNA polymerase. PCR amplification consisted of an initial denaturation step of 98 °C for 30 s, followed by 35 cycles of 94 °C for 30 s, 58 °C for 30 s, 72 °C for 90 s, with a final cycle of 72 °C for 5 min. Amplicons were cleaned using ExoSAP-IT (Affimetrix/USB, Santa Clara, CA, USA) and used as templates for direct DNA sequencing. Samples for DNA sequencing were prepared using a BigDye Terminator Cycle Sequencing Ready Reaction kit (Life Technologies, Foster City, CA, USA) by primer walking and run on a 3730 × l DNA Analyzer (Life Technologies, Carlsbad, CA USA).

### Population genetics analysis

The *T. saginata cox*1 sequences were aligned using ClustalW [19] and a phylogenetic tree was constructed using the maximum likelihood algorithm implemented in MEGA 6. Tamura-Nei (TN93 + G + I) was used as the best-fit substitution model [20] and support for groupings within the tree was evaluated by bootstrapping with 1,000 replicates [21]. A network analysis was performed using the median-joining method included in the Network software version 5.0.0.0 (Fluxus Technology Ltd., www.fluxus-engineering.com).

The following measures for population genetics based on complete *T. saginata cox*1 sequences from this study and GenBank were calculated: genetic variability at polymorphic sites between populations (*S*), haplotype numbers (*h*), haplotype diversity (*Hd*), nucleotide diversity (*π*), population mutation rates based on the number of segregation sites (*θω*) and mean number of pairwise differences (*θπ*) [22–25] were calculated using DnaSP version 4.0 [26]. Statistical analysis to distinguish between DNA sequences evolving randomly (neutrality) and those evolving under a non-random process was done using Tajima’s D [27] and Fu’s *Fs* tests [28].

## Results

All of the 51 taeniid isolates collected in Lao PDR and northeastern Thailand were identified as *T. saginata* based on the diagnostic nucleotide (= adenine) at position 723 of *cox*1 [29], although slight intra-population variation was observed. BLAST searches also revealed that the 51 sequences showed high homology (> 99%) to *T. saginata* sequences deposited in GenBank. The complete sequence analysis revealed that 15 and 8 haplotypes were detected in 30 and 21 *T. saginata* isolates from Lao PDR and northeastern Thailand, respectively. Fifty-three haplotypes were identified in 98 sequences, which included 47 sequences from GenBank. The haplotypes, haplotype frequencies, and accession numbers for the 98 *T. saginata cox*1 sequences are presented in Table 1.

**Table 1:** *Taenia saginata* haplotypes and their frequencies in Lao PDR, Thailand and other countries

Haplotype	Haplotype frequency	Sample (Country) code/GenBank accession number
H1	1	T2 (THA)/KY290351
H2	1	T3 (THA)/KY290352
H3	8	T4, T6, T7, T8, T13, T16, T17, T19 (THA)/KY290353
H4	1	T5 (THA)/KY290354
H5	1	T9 (THA)/KY290355
H6	4	T10, T20, T21, T22 (THA)/KY290356
H7	3	T11, T14, T24 (THA)/KY290357
H8	2	T12, T23 (THA)/KY290358
H9	1	L1.1(LAO)^a^/KY290359
H10	1	L1.2 (LAO)^a^/KY290360
H11	13	L1.3^a^, L2.1^b^, L2.3^b^, L4, L8, L10, L17, L18, L19, L31, L65.1, L67, L71 (LAO)/KY290361
H12	1	L2.2^b^ (LAO)/KY290362
H13	1	L2.4^b^ (LAO)/KY290363
H14	1	L3 (LAO)/KY290364
H15	1	L5 (LAO)/KY290365
H16	3	L6, L12, L73 (LAO)/KY290366
H17	1	L7 (LAO)/KY290367
H18	2	L9, L11 (LAO)/KY290368
H19	1	L13 (LAO)/KY290369
H20	1	L14 (LAO)/KY290370
H21	1	L15 (LAO)/KY290371
H22	1	L16 (LAO)/KY290372
H23	1	L65.2 (LAO)/KY290373
H24	7	AB107240 (IDN), AB465245 (ETH), AB465246 (KOR), AB465247 (THA), AB465248 (THA), AB644391 (JPN), AB820291 (DJI)
H25	1	AB984348 (CHN)
H26	1	AB821273 (ETH)
H27	1	AB465243 (ECU)
H28	1	AY684274 (Africa, not specified)
H29	10	AB107244 (THA), AB275143 (KHM), AB107242 (BEL), AB107247 (CHN), AB465231 (THA), AB465233 (THA), AB465234 (THA), AB465241 (KHM), AB533168 (CHN), AB465232 (THA)
H30	1	AB107239 (CHN)
H31	1	AB984351 (CHN)
H32	1	AB645845 (THA)
H33	1	AB465242 (THA)
H34	1	AB465240 (IDN)
H35	1	AB271695 (MNG)
H36	1	AB107238 (ECU)
H37	2	AB107237 (BRA), AB465238 (BRA)
H38	1	AB533169 (CHN)
H39	1	AB533172 (CHN)
H40	1	AB107243 (NPL)
H41	1	AB107241 (ETH)
H42	1	AY195858 (Africa, not specified)
H43	1	AB533171 (CHN)
H44	1	AB465237 (ETH)
H45	1	AB066495 (CHN)
H46	1	AB984350 (CHN)
H47	1	AB984347 (CHN)
H48	1	AB465239 (THA)
H49	1	AB984346 (CHN)
H50	2	AB465235 (THA), AB465236 (THA)
H51	1	AB533173 (THA)
H52	1	AB107246 (BRA)
H53	1	AB107245 (THA)

^a, b^Three (L1.1-L1.3) and four (L2.1-L2.4) tapeworms were expelled from two patients in Lao PDR. Such a situation is not common, but it is possible to find taeniosis patients with two and more adult tapeworms in endemic areas in Lao PDR and Thailand [34]

*Note*: For GenBank sequences (H24-H53), the numbers in the “Haplotype frequency” column indicate the numbers analyzed, not haplotype frequency

*Abbreviations*: *BEL* Belgium, *BRA* Brazil, *CHN* China, *DJI* Djibouti, *ECU* Ecuador, *ETH* Ethiopia, *IDN* Indonesia, *JPN* Japan, *KHM* Cambodia, *KOR* Korea, *LAO* Lao PDR, *MNG* Mongolia, *NPL* Nepal, *THA* Thailand

Phylogenetic tree analysis using the 98 sequences indicated that global isolates of *T. saginata* could be genetically divided into five groups: A, B, D and two subgroups (C1 and C2) (see Additional file 1: Figure S1). Groups B and C1 diverged at the base of the phylogenetic tree. Although Group A branched off from Group B, the bootstrap values were not high. Group D diverged from subgroup C2. Groups A and B were composed of *T. saginata* populations from Lao PDR and northeastern Thailand. Subgroups C1 and C2 consisted of isolates from western Thailand and other geographical locations around the world. Group D was composed of a few *T. saginata* from western Thailand, which included Bangkok. It was unclear whether the patients had acquired the *T. saginata* infection in Bangkok or if they were just living in Bangkok. There was no common haplotype for *T. saginata* populations from western Thailand and other geographically different countries.

**Fig. 1:**
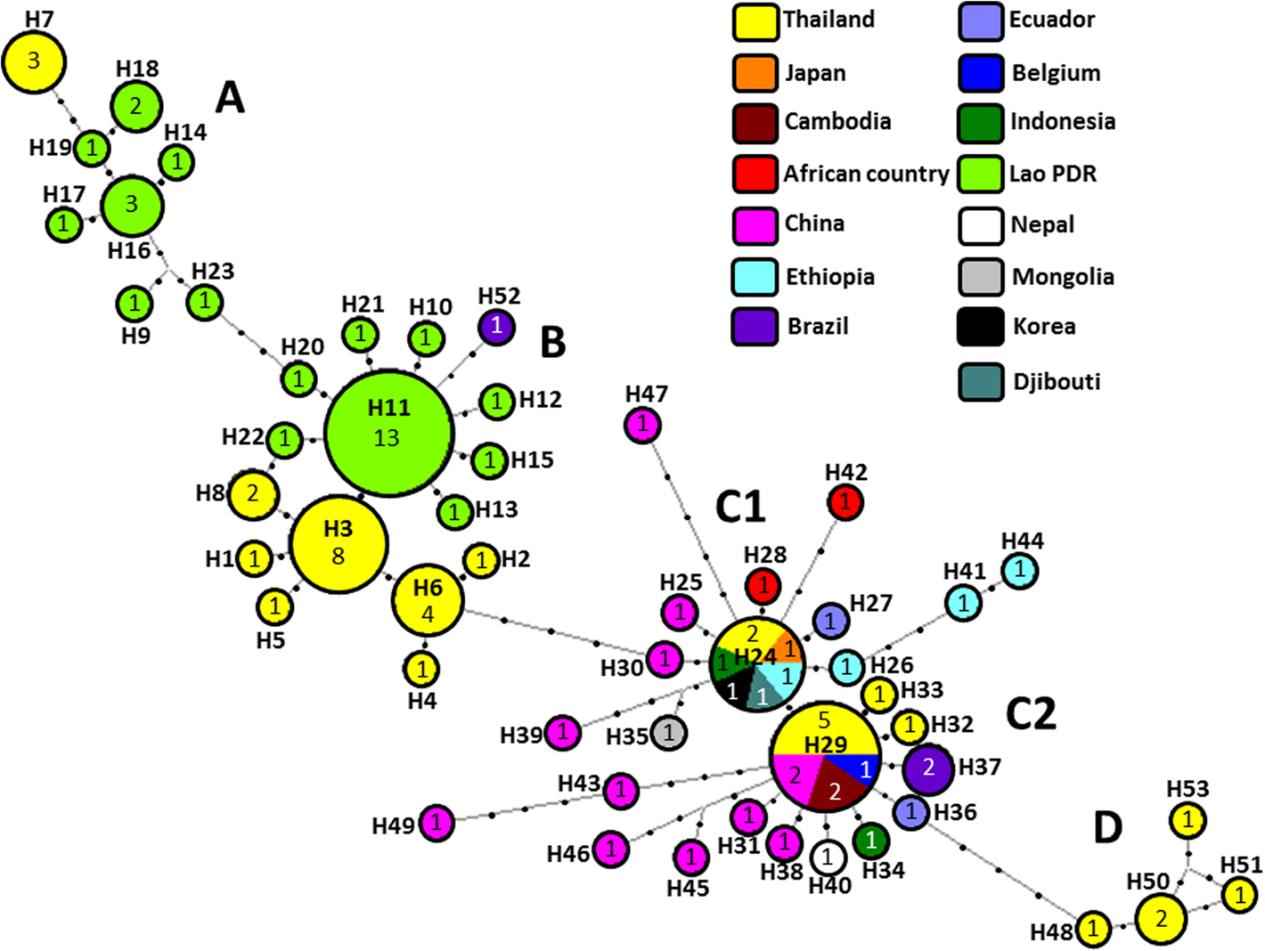
Haplotype network of *Taenia saginata* from Lao PDR, Thailand and other countries. The size of the circles corresponds to the frequency of the haplotypes identified. Dots denote the number of nucleotide substitutions. Countries where *T. saginata* was collected are color-coded

In addition to phylogenetic tree analysis, the median-joining network also revealed that *T. saginata* isolates from around the world could be divided into five groups. *Taenia saginata* from Lao PDR and northeastern Thailand were separated in two scattered groups, Groups A and B, which were linked to each other (Fig. 1). Group A was composed of seven haplotypes (H9, H14, H16-H19 and H23) from Lao PDR and one haplotype (H7) from northeastern Thailand. Group B formed a firework-shaped structure around the predominant haplotypes (Lao H11 and Thai H3). Although one haplotype (H52) from Brazil was included in Group B, the phylogenetic relationship between *T. saginata* from these countries is unknown. Subgroup C1 was composed of a dominant haplotype (H24) from western Thailand, Japan, Korea, Indonesia, Djibouti and Ethiopia, as well as other haplotypes from China (H25, H30, H39 and H47), Mongolia (H35), Ecuador (H27), Ethiopia (H26, H41 and H44), and unspecified African countries (H28 and H42). Haplotype H29 was predominant in subgroup C2 and was composed of isolates from western Thailand, Cambodia, China and Belgium, and other haplotypes were from China (H31, H38, H43, H45, H46 and H49), western Thailand (H32 and H33), Indonesia (H34), Nepal (H40), Ecuador (H36) and Brazil (H37). Subgroups C1 and C2 were closely linked and formed firework-type structures around the two predominant haplotypes H24 and H29, respectively. Interestingly, Group D was composed of a few haplotypes (H48, H50, H51 and H53) from Kanchanburi Province, western Thailand and Bangkok, and *T. saginata* from northeastern and western Thailand were genetically distinct populations.

To estimate the genetic diversity in *T. saginata* populations from Lao PDR and northeastern Thailand, several population genetics indices based on the complete *cox*1 sequences are presented in Table 2. The number of *T. saginata* haplotypes identified from Lao PDR and northeastern Thailand was 15 and 8, respectively, and the number of segregation sites was 20 (1.23%) in Lao PDR and 10 (0.67%) in northeastern Thailand. The overall number of haplotypes was 53 out of 98 sequences and 57 sites (3.79%) were segregated. The haplotype diversity (*Hd*) was as high as 0.926 ± 0.038 and 0.819 ± 0.064 in *T. saginata* populations from Lao PDR and northeastern Thailand, respectively. The global *Hd* (0.968 ± 0.020) was also high in *T. saginata* including the western Thai population (0.868 ± 0.076). In contrast, the nucleotide diversity (*π*) was as low as 0.00252 ± 0.0003 and 0.0015 ± 0.00034 in *T. saginata* from Lao PDR and northeastern Thailand, respectively. Overall the nucleotide diversity of haplotypes of *T. saginata* worldwide was as low as 0.00381 ± 0.00021, including the western Thai population (0.0236 ± 0.00041). *θπ* was less than *θω* in *T. saginata* populations from Lao PDR and northeastern Thailand. Neutrality in *T. saginata* populations from Lao PDR and northeastern Thailand was not significant by Tajima’s D test (-0.66964, *P* = 0.10, and -0.66053, *P* = 0.10) and Fu’s *Fs* test (-1.670, *P* = 0.094). However, a pairwise Fu’s *Fs* test showed a significant value (-10.078, *P* < 0.0001) in the Lao population. In haplotypes overall, Tajima’s D value (-2.31270, *P* = 0.01) and a Fu’s *Fs* value (-21.042, *P* < 0.0001) were significant.

**Table 2:** Genetic diversity of *Taenia saginata* populations from Lao PDR, Thailand and other countries

Populations	No. of samples	*h*	*S* (%)	*Hd*	*π*	*θɷ*	*θπ*	Tajima’s *D* (*P*-value)	Fu’s *Fs* (*P-*value)
Lao PDR	30	15	20 (1.23)	0.926 ± 0.038	0.00252 ± 0.00030	5.048	4.078	-0.66964 (*P* = 0.10)	-10.078 (*P* < 0.0001)*
Northeastern Thailand	21	8	10 (0.67)	0.819 ± 0.064	0.0015 ± 0.00034	2.780	2.248	-0.66053 (*P* = 0.10)	-1.670 (*P* = 0.094)
Western/Central Thailand	14	8	11 (0.68)	0.868 ± 0.076	0.00236 ± 0.00041	3.459	3.824	0.41614 (*P* = 0.10)	-1.195 (*P* = 0.139)
Northeastern Thailand and Lao PDR	51	24	21 (1.40)	0.915 ± 0.025	0.00226 ± 0.00023	4.667	3.402	-0.86683 (*P* = 0.10)	-14.288 (*P* < 0.0001)*
World except for Lao PDR and Thailand	33	25	44 (2.72)	0.968 ± 0.020	0.00245 ± 0.00037	10.841	3.962	-2.31270 (*P* = 0.01)*	-21.042 (*P* < 0.0001)*
All samples	98	53	57 (3.80)	0.960 ± 0.010	0.00381 ± 0.00021	11.053	5.726	-1.60729 (*P* = 0.10)	-43.930 (*P* < 0.0001)*

*Abbreviations*: *h* haplotype numbers, *S* number of segregation sites, *Hd* haplotype diversity, *π* nucleotide diversity, *θɷ* Watterson’s theta based on *S*, *θπ* theta based on *π*

*P < 0.05

## Discussion

In this study, we present molecular evidence of *T. saginata* infection in people in central Lao PDR and northeastern Thailand. We explored genetic diversity based on the complete *cox*1 sequences of *T. saginata* populations from these regions and also included *T. saginata* data available from GenBank. To our knowledge, this is the first molecular identification and genetic diversity analysis of *T. saginata* from Lao PDR.

Phylogenetic tree and haplotype network analysis both revealed that *T. saginata* could be genetically divided into five groups and that *T. saginata* populations from Lao PDR and northeastern Thailand belonged to Group A and Group B. Subgroup C1 included the western Thai population, and Group D was also composed of western Thai isolates, but was differentiated from subgroup C2. *Taenia saginata* populations from northeastern and western Thailand are geographically and genetically isolated and, to date, there is no evidence that they are sympatrically distributed in Thailand. It has been suggested that haplotype H11 may be an ancestral haplotype and that other haplotypes in the Lao population have diverged from it (Fig. 1). Subgroups C1 and C2 are composed of the predominant haplotypes (H24 and H29) and many haplotypes with low substitution rates. The predominant haplotypes were also detected in China, suggesting that either haplotype (H24 or H29) may be an ancestral haplotype in these areas. In contrast, the phylogenetic relationships between Lao (H11) and Brazilian haplotypes (H52), the Thai *T. saginata* (H48) and Ecuadorian haplotypes (H36), and many of the global *T. saginata* isolates are unclear in Subgroups C1 and C2. Further study using other molecular markers is necessary to explain the complex phylogenetic relationships that exist between these *T. saginata* populations.

The *h* and *π* values in *T. saginata* from northeastern Thailand were lower than those from the Lao population. *θπ* was less than *θω* in *T. saginata* populations from Lao PDR and northeastern Thailand. While the results of Tajima’s D test were not significant in the Lao and northeastern Thai populations, Fu’s *Fs* test showed that the Lao population is genetically differentiated (-10.078, *P* < 0.0001). This discrepancy between the two statistical tests is considered to explain why more haplotypes were detected compared with the number of segregated sites, and indicated that the genetic diversity in the Lao population was high. However, compared with other global *T. saginata* isolates, the degree of genetic differentiation in the Lao and northeastern Thai populations was low. This also supports the finding that Groups A and B are linked to each other, in particular, haplotype H6 in Group B was linked to haplotype H30 from China in Subgroup C1. Group B formed a firework-like structure, which was differentiated by the small base substitution rate around predominant haplotypes H3 and H11. *Taenia saginata* populations are dispersed in different geographic localities where genetic variation is influenced by the genetic bottleneck effect that arises from decreases in the population size. However, because the sample-size that was examined in this study was small, further study using more samples from around the world is necessary to more accurately clarify the effect of such genetic events in geographically different populations.

Anantaphruti et al. [4] identified 14 haplotypes in 73 *T. saginata* isolates from northern and northeastern Thailand based on partial *cox*1 sequences (924 bp). In their study, haplotypes A and B were predominant, and haplotype A was found in ten countries, including Thailand. These authors proposed that haplotype A shared the same ancestor as *T. saginata* populations from around the world. However, the findings of our network analysis differ from those in their report because the complete *cox*1 sequence of their haplotype B is not available, and the haplotype in our study corresponding to their haplotype B has not been identified. Nonetheless, their haplotype A is identical to haplotype H29 in our study, but it was only found in Thailand, Belgium, China and Cambodia as shown in Fig. 1. This discrepancy can probably be attributed to the different lengths of *cox*1 sequences because we analyzed the complete sequence (1,620 bp), whereas only 924 bp was analyzed previously [4].

The fact that genetically different *T. saginata* populations are distributed in Lao PDR and Thailand is probably closely associated with the artificial movement of cattle as an intermediate host. The artificial movement of cattle may have played an important role in the dispersal of liver fluke *Fasciola* species in Asia [30, 31]. To explain the geographically distinct nature of the *T. saginata* populations in Lao PDR and Thailand, the following scenario is possible: *T. saginata* originating in southern provinces of China might have been transmitted, via northwestern Thailand and Myanmar, to the western regions of Thailand (western route), and via northern Lao PDR and Vietnam to northeastern regions of Thailand (eastern route). Network analysis using more *T. saginata* specimens from different Asian countries, including China, is necessary to verify this scenario and to provide a more detailed estimate of the genetic diversity of *T. saginata*. The sample size in population genetics analysis can modulate the neutrality [32] and the size of the target gene can also interfere with population genetic indices as discussed above.

While taeniosis has a low impact on human health, it is an important zoonosis that can cause serious economic losses in cattle [7–11]. Consequently, understanding the population genetics of the parasite will help to elucidate transmission patterns and to develop control measures [33].

## Conclusions

*Taenia saginata* from Lao PDR and northeastern Thailand showed high haplotype diversity, but the degree of genetic differentiation was low. Lao and northeastern Thai populations were also found to be genetically distinct form the *T. saginata* population in western Thailand, suggesting that *T. saginata* is spread by different transmission routes in Southeast Asia.

## Additional file

Additional file 1: Figure S1. The maximum likelihood tree constructed from *cox*1 sequences of *T. saginata*. Bootstrap scores (percentages of 1,000 replications) are presented at each node. Samples used to obtain the nucleotide sequences in this study are represented with sample codes in bold (KY290351–KY290373, see Table 1). Sequence data from GenBank are shown with accession numbers, country codes and haplotype names. Scale-bar indicates the number of nucleotide substitutions/site. (TIF 5144 kb)
