# Supplementary figures and images for: Genetic diversity of Taenia saginata (Cestoda: Cyclophyllidea) from Lao People’s Democratic Republic and northeastern Thailand based on mitochondrial DNA
Source: Parasit Vectors. 2017 Mar 11;10:141. doi: 10.1186/s13071-017-2079-7 (PMC5346190; doi:10.1186/s13071-017-2079-7)

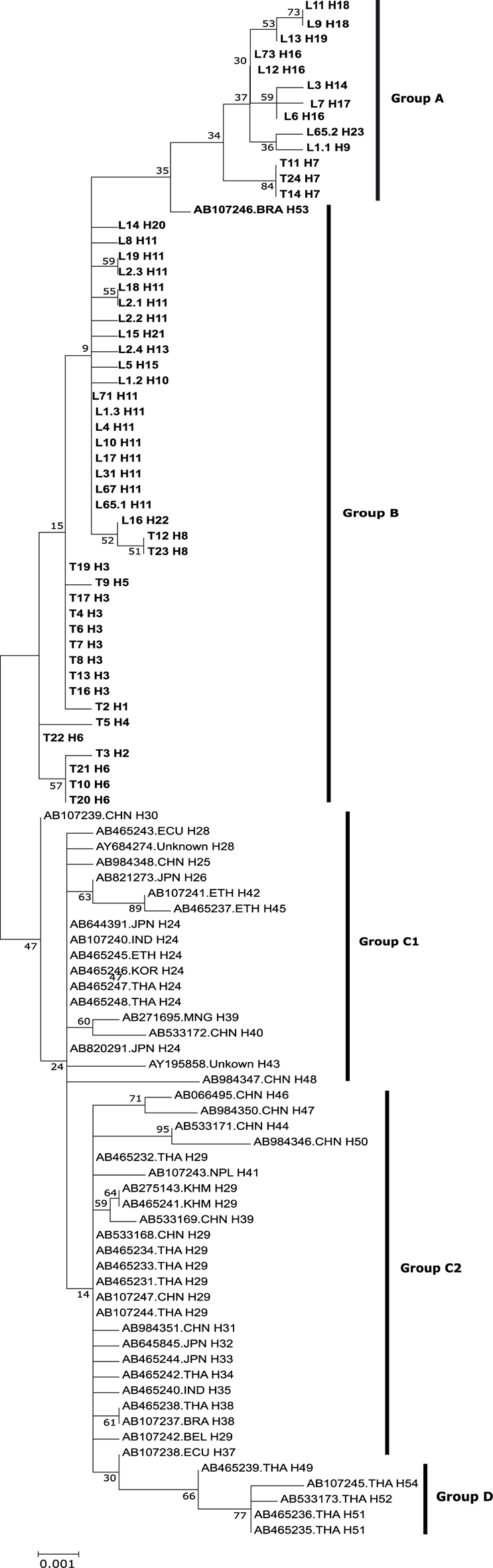

Supplement: Additional file 1: Figure S1. — The maximum likelihood tree constructed from cox1 sequences of T. saginata. Bootstrap scores (percentages of 1,000 replications) are presented at each node. Samples used to obtain the nucleotide sequences in this study are represented with sample codes in bold (KY290351–KY290373, see Table 1). Sequence data from GenBank are shown with accession numbers, country codes and haplotype names. Scale-bar indicates the number of nucleotide substitutions/site. (TIF 5144 kb) [file 13071_2017_2079_MOESM1_ESM.tif]
